# 

**Published:** 1874-05

**Authors:** 


### CONTENTS:

ORIGINAL COMMUNICATIONS:
ARTICLE I.—Cerebro-Spinal Meningitis, or Spotted Fever. By J. S. Whitmire, M.D. .... 257
ART. II.—Waxy Kidney—with Case. By M. W. Wood, M.D. .... 273
ART. III.—Review of the Article entitled "Infants and their Food." By G. Newkirk, M.D. .... 284
ART. IV.—Review of April No. Chicago Medical Journal. By Z. C. McElroy, M.D. .... 290

PROGRESS IN MEDICAL SCIENCES:
ART. I.—Progress of Surgery. By Jno. E. Owens, M.D. .... 297
ART. II.—Progress of Syphilology. By J. N. Hyde, M.D. .... 303
EDITORS' BOOK TABLE .... 311
EDITORIAL .... 317
NOTICE—Meeting of Amer. Med. Association .... 317
GRADUATES OF RUSH MED. COLLEGE, 1873-4 .... 319

